# COVID19 biomarkers: What did we learn from systematic reviews?

**DOI:** 10.3389/fcimb.2022.1038908

**Published:** 2022-12-13

**Authors:** Sabina Semiz

**Affiliations:** College of Medicine and Health Sciences, Khalifa University, Abu Dhabi, United Arab Emirates

**Keywords:** SARS-CoV-2, biomarkers, prognosis, severity, mortality

## Abstract

The coronavirus disease 2019 (COVID19) pandemic continues to represent a substantial public health concern. It can rapidly progress to severe disease, with poor prognosis and a high mortality risk. An early diagnosis and specific prognostic tools can help healthcare providers to start interventions promptly, understand the likely prognosis and to identify and treat timely individuals likely to develop severe disease with enhanced mortality risk. Here we focused on an impressive set of systematic reviews and meta-analyses that were performed since the start of the COVID19 pandemic and summarized their results related to the levels of hematologic, inflammatory, immunologic biomarkers as well as markers of cardiac, respiratory, hepatic, gastrointestinal and renal systems and their association with the disease progression, severity and mortality. The evidence outlines the significance of specific biomarkers, including inflammatory and immunological parameters (C-reactive protein, procalcitonin, interleukin-6), hematological (lymphocytes count, neutrophil-to-lymphocyte ratio, D-dimer, ferritin, red blood cell distribution width), cardiac (troponin, CK-MB, myoglobin), liver (AST, ALT, total bilirubin, albumin) and lung injury (Krebs von den Lungen-6) that can be used as prognostic biomarkers to aid the identification of high-risk patients and the prediction of serious outcomes, including mortality, in COVID19. Thus, these parameters should be used as essential tools for an early risk stratification and adequate intervention in improving disease outcomes in COVID19 patients.

## 1 Introduction

As the number of confirmed cases of the Coronavirus disease 2019 (COVID19) overpassed 630 million and almost 7 million people lives were claimed globally due to this devastating disease, significant efforts are being made to learn how to efficiently prevent, control and treat this evolving viral infection and its progression to the serious stage of disease. COVID19 demonstrates a clinically diverse manifestation ranging from asymptomatic carriers to fulminant disease characterized by severe pneumonia, respiratory failure, sepsis or multiple organ failure often associated with detrimental outcomes and poor survival. It was reported that about 20% of hospitalized patients with COVID19 experience severe symptoms requiring intensive care (Wiersinga et al., 2020) and that the overall mortality rate was about 18% (Shoar et al., 2020).

Since the start of the Coronavirus Disease (COVID19) pandemic in 2019, intensive research has been initiated to better understand the mechanisms of the severe acute respiratory syndrome coronavirus 2 (SARS-CoV-2) infection and disease development, to develop an effective preventive and treatment options, as well as to predict and diagnose this devastating disease more efficiently in order to prevent its serious, often fatal, outcomes. The COVID19 pandemic demonstrated variable clinical progression, from some patients remaining asymptomatic to the majority of the patients presenting with cough, fever, shortness of breath, and sore throat (Struyf et al., 2022). However, as this recent update of the Cochrane systematic review concluded, most of the reviewed individual symptoms have poor diagnostic accuracy for COVID19 (Struyf et al., 2022). The recent evidence of using wearable devices to record the subtle changes in physiological parameters, including skin temperature, heart and respiratory rate, although in an early phase, demonstrated the potential for early detection of SARS-CoV-2 infection (Mitratza et al., 2022). The presence of anosmia or ageusia was also suggested as a red flag for COVID19, while the presence of fever or cough may be useful to identify individuals for further testing (Struyf et al., 2022). Furthermore, SARS-CoV-2 infection can cause mild to moderate COVID19 disease and most patients recover from the disease, while others develop COVID19 pneumonia and other pulmonary, cardiac and neurologic complications leading some patients to require intensive care support and, in some cases, causing death, especially in older adults. Clinical history and a laboratory profile may help identify COVID19 patients with a higher risk of mortality. On the other side, a recent systematic review and meta-analysis reported that about 16% of confirmed COVID19 patients are asymptomatic and that about 50% of the patients with no symptoms at detection time will have symptoms later (He et al., 2021). These asymptomatic COVID19 patients could have abnormal laboratory manifestations, which can be used as screening strategies to identify asymptomatic infection.

Thus, there is a clear and urgent need to select specific diagnostic and prognostic tools which could identify severe cases and predict outcomes of SARS-CoV-2 infection. Various biomarkers are currently under investigation to assess their potential use in diagnosis and prognosis of COVID19 and to understand how levels of different biomarkers of COVID19 vary during the course of the disease. Strikingly, a high number of systematic reviews and meta-analysis have been performed since the start of COVID19 pandemic which could lead to the identification of potential biomarkers that can be employed in predicting the outcome of SARS-CoV-2 infection. The aim of this work was to summarize the findings of these systematic reviews and meta-analyses investigating the routine laboratory biomarkers measurement in COVID-19 patients and evaluating their potential role to predict clinical outcomes, the severity and mortality of COVID19 disease, thus leading to more efficient COVID-19 diagnosis, treatment and overall positive disease outcomes.

## 2 Methods

A literature search in PubMed database was conducted till August 31, 2022 by using the terms such as biomarkers, diagnosis, prognosis, predictive, severity and mortality, alongside COVID‐19 or SARS-CoV-2 or coronavirus. From this search, only systematic review and meta-analysis articles were retrieved for further review. The clinical data of COVID19 patients, related to symptoms, diagnosis, prognosis, disease outcomes, the organ systems affected by diseases and laboratory parameters/biomarkers used in diagnosis and prognosis of COVID19 are summarized here.

## 3 Biomarkers of COVID19 severity and mortality

### 3.1 COVID19 severity

Increased COVID19 severity and/or mortality was found to be associated with age over 55 years, multiple preexisting conditions, extensive lung involvement, hypoxia, abnormalities of diverse laboratory findings and biomarkers of multiple organ dysfunction (Gallo Marin et al., 2021). As shown in **Table 1**, findings from systematic reviews and meta-analyses demonstrated that several routine laboratory tests were associated with disease severity in patients with COVID19. Severe disease was strongly associated with fever, cough, pneumonia, lymphopenia, increased levels of C-reactive protein (CRP), aspartate aminotransferase (AST) and alanine aminotransferase (ALT) activity, older age and male gender (Borges Do Nascimento et al., 2020; Zhang et al., 2020b). Additional systematic reviews demonstrated that severe disease was also associated with higher white blood cells (WBC) and neutrophils counts, CRP, lactate dehydrogenase (LDH), AST and ALT activity, D-dimer, as well as with low lymphocyte and platelet counts, hemoglobin levels, and prolonged prothrombin time (PT) (Alnor et al., 2020; Elshazli et al., 2020). Furthermore, higher levels of CRP, serum amyloid A (SAA), interleukin-6 (IL6), LDH, neutrophil-to-lymphocyte ratio (NLR), D-dimer, cardiac troponin, and renal biomarkers were also shown in patients with severe complications of COVID19 infection, while lymphocytes and platelet count demonstrated lower levels in severe patients as compared to their nonsevere counterparts (Kermali et al., 2020). Furthermore, a recent systematic review and meta-analysis (Rostami et al., 2021) showed elevated levels of fibrinogen, fibrinogen (or fibrin) degradation products (FDPs), and D-dimer in severe COVID19 as compared with patients with nonsevere form of this disease, suggesting that a continuous rise in levels of fibrinogen, D-dimer, and FDP may predict critical prognosis in patients with COVID19 (Rostami et al., 2021).

**Table 1:** Laboratory biomarkers associated with immune system, injury of different organ systems, disease severity and mortality in patients with COVID19.

System	Laboratory Biomarkers	COVID19 Outcome (Mortality/Severity)	Change	References(systematic reviews/meta-analysis)
**Immune System**	CRP	Mortality & Severity	↑	(Alnor et al., 2020; Zhang et al., 2020b; Tian et al., 2020; Kermali et al., 2020; Borges Do Nascimento et al., 2020; Elshazli et al., 2020; Walker et al., 2020; Yamada et al., 2020; Huang et al., 2020; Mudatsir et al., 2020; Malik et al., 2021; Iwamura et al., 2021; Hariyanto et al., 2021; Lim et al., 2021; Melo et al., 2021; Chaudhary et al., 2021; Ikeagwulonu et al., 2021; Yitbarek et al., 2021; Mosquera-Sulbaran et al., 2021; Alzahrani and Al-Rabia, 2021; Bhowmik et al., 2022; Cao et al., 2022)
	IL4	Severity	↑	(Cao et al., 2022)
	IL6	Mortality & Severity	↑	(Kermali et al., 2020; Tian et al., 2020; Li et al., 2020c; Elshazli et al., 2020; Walker et al., 2020; Coomes and Haghbayan, 2020; Mudatsir et al., 2020; Iwamura et al., 2021; Melo et al., 2021; Udomsinprasert et al., 2021; Chaudhary et al., 2021; Zawawi et al., 2021; Liu et al., 2021a; Alzahrani and Al-Rabia, 2021; Luo et al., 2022; Jafrin et al., 2022; Cao et al., 2022)
	IL8	Severity	↑	(Zawawi et al., 2021; Liu et al., 2021a)
	IL10	Mortality & Severity	↑	(Udomsinprasert et al., 2021; Zawawi et al., 2021; Liu et al., 2021a; Jafrin et al., 2022; Cao et al., 2022)
	TNF	Severity	↑	(Zawawi et al., 2021; Liu et al., 2021a)
	C3	Mortality & Severity	↓	(Zinellu and Mangoni, 2021b)
	C4	Mortality & Severity	↓	(Zinellu and Mangoni, 2021b)
	CD3+ T cells	N/A	↓	(Iwamura et al., 2021)
	CD4+ T cells	N/A	↓	(Iwamura et al., 2021)
	aPL	Severity	↑	(Taha and Samavati, 2021)
	IFN-α	N/A	↑	(Da Silva et al., 2021)
	Vitamin D	N/A	↓	(Munshi et al., 2021)
	PCT	Mortality & Severity	↑	(Zheng et al., 2020; Huang et al., 2020; Zare et al., 2020; Malik et al., 2021; Melo et al., 2021; Iwamura et al., 2021; Hariyanto et al., 2021; Ahmed et al., 2021a)
	Serum amyloid A	Severity	↑	(Kermali et al., 2020; Cao et al., 2022)
	ESR	Severity	↑	(Mudatsir et al., 2020; Iwamura et al., 2021)
	Prealbumin	Mortality & Severity	↓	(Zinellu and Mangoni, 2021c)
	Calprotectin	Severity	↑	(Udeh et al., 2021)
	Presepsin	Severity	↑	(Ahmed et al., 2021b)
	OAS1	N/A	↓	(Luo et al., 2022)
Pediatric Multisystem Inflammatory Syndrome (PMIS)	CRP	Mortality & Severity	↑	(Yasuhara et al., 2020; Abrams et al., 2020; Zhao et al., 2021c; Tang et al., 2021)
	D-dimer	Mortality & Severity	↑	(Yasuhara et al., 2020; Zhao et al., 2021c; Tang et al., 2021)
	PCT	Mortality & Severity	↑	(Huang et al., 2020; Tang et al., 2021)
	Lymphocytes count	Mortality & Severity	↓	(Yasuhara et al., 2020; Zhao et al., 2021c; Tang et al., 2021)
	Platelet count	Mortality & Severity	↓	(Zhao et al., 2021c)
	Neutrophil count	Mortality & Severity	↑	(Zhao et al., 2021c; Tang et al., 2021)
	WBC count	Severity	↑	(Zhao et al., 2021c)
	Ferritin	Mortality & Severity	↑	(Zhao et al., 2021c; Tang et al., 2021)
	ESR	Severity	↑	(Zhao et al., 2021c; Tang et al., 2021)
	IL6	Severity	↑	(Abrams et al., 2020; Tang et al., 2021)
	Fibrinogen	Severity	↑	(Abrams et al., 2020)
	BNP	Severity	↑	(Zhao et al., 2021b)
	LDH	Mortality & Severity	↑	(Zhao et al., 2021c)
**Hematology**	WBC count	Mortality & Severity	↑	(Alnor et al., 2020; Yamada et al., 2020; Elshazli et al., 2020; Mudatsir et al., 2020; Xiang et al., 2021b; Chua et al., 2021; Liu et al., 2021a; Suklan et al., 2021; Melo et al., 2021)
	Neutrophil count	Mortality & Severity	↑	(Alnor et al., 2020; Elshazli et al., 2020; Chua et al., 2021; Liu et al., 2021a; Lim et al., 2021; Suklan et al., 2021; Melo et al., 2021; Cao et al., 2022)
	Lymphocytes count	Mortality & Severity	↓	(Alnor et al., 2020; Kermali et al., 2020; Borges Do Nascimento et al., 2020; Figliozzi et al., 2020; Zhang et al., 2020b; Huang and Pranata, 2020; Suklan et al., 2021; Malik et al., 2021; Xiang et al., 2021b; Iwamura et al., 2021; Lim et al., 2021) (Zhang et al., 2020b; Figliozzi et al., 2020; Malik et al., 2021; Xiang et al., 2021b; Melo et al., 2021) (Alnor et al., 2020; Kermali et al., 2020; Borges Do Nascimento et al., 2020; Huang and Pranata, 2020; Mudatsir et al., 2020; Suklan et al., 2021; Malik et al., 2021; Melo et al., 2021; Iwamura et al., 2021; Cao et al., 2022)
	NLR	Mortality & Severity	↑	(Kermali et al., 2020; Li et al., 2020b; Alkhatip et al., 2021; Ulloque-Badaracco et al., 2021b; Simadibrata et al., 2021; Sarkar et al., 2022a; Sarkar et al., 2022b; Bhowmik et al., 2022; Parthasarathi et al., 2022)
	Platelet count	Mortality & Severity	↓	(Alnor et al., 2020; Kermali et al., 2020; Terpos et al., 2020; Suklan et al., 2021; Malik et al., 2021; Xiang et al., 2021b; Pranata et al., 2021)
	Hemoglobin	Severity	↓	(Alnor et al., 2020)
	Ferritin	Mortality & Severity	↑	(Taneri et al., 2020; Huang et al., 2020; Melo et al., 2021; Chaudhary et al., 2021; Kaushal et al., 2022)
	Fibrinogen	Severity	↑	(Zhang et al., 2020a; Rostami et al., 2021; Chaudhary et al., 2021)
	FDPs	Severity	↑	(Rostami et al., 2021)
	D-dimer	Mortality & Severity	↑	(Alnor et al., 2020; Kermali et al., 2020; Tian et al., 2020; Figliozzi et al., 2020; Zheng et al., 2020; Paliogiannis et al., 2020; Elshazli et al., 2020; Terpos et al., 2020; Ji et al., 2020; Zhang et al., 2020a; Rostami and Mansouritorghabeh, 2020; Duz et al., 2020; Simadibrata and Lubis, 2020; Sakka et al., 2020; Lima et al., 2020; Rostami et al., 2021; Malik et al., 2021; Chua et al., 2021; Liu et al., 2021a; Xiang et al., 2021a; Zhao et al., 2021a; Varikasuvu et al., 2021; Hariyanto et al., 2021; Nugroho et al., 2021; Woller et al., 2022)
	PT	Severity	↑	(Elshazli et al., 2020; Terpos et al., 2020; Zhang et al., 2020a; Liu et al., 2021a; Xiang et al., 2021a)
	RDW	Severity	↑	(Lee et al., 2021; Zinellu and Mangoni, 2021a)
	ABO	N/A	↑	(Luo et al., 2022)
**Endothelial dysfunction**	MR-proADM	Severity	↑	(Lampsas et al., 2021)
	E-selectin	Severity	↑	(Lampsas et al., 2021)
	VCAM-1	Severity	↑	(Lampsas et al., 2021)
	VWF-Ag	Severity	↑	(Lampsas et al., 2021; Andrianto et al., 2021)
	Ang-2	Severity	↑	(Lampsas et al., 2021)
	T-PA	Severity	↑	(Andrianto et al., 2021)
	PAI-1	Severity	↑	(Andrianto et al., 2021)
	sTM	Severity	↑	(Andrianto et al., 2021)
**Cardiac Injury**	Cardiac troponin	Mortality & Severity	↑	(Kermali et al., 2020; Tian et al., 2020; Vakhshoori et al., 2020b; Toraih et al., 2020; Parohan et al., 2020; Shoar et al., 2020; Rathore et al., 2021; Alzahrani and Al-Rabia, 2021; Wibowo et al., 2021)
	TnT	Mortality & Severity	↑	(Alzahrani and Al-Rabia, 2021)
	Tnl	Mortality & Severity	↑	(Li et al., 2020a; Parohan et al., 2020; Li et al., 2020c; Zheng et al., 2020; Ma et al., 2021; Zhu et al., 2021)
	NT-proBNP	Mortality & Severity	↑	(Li et al., 2020a; Ramadan et al., 2021; Dalia et al., 2021)
	BNP	Mortality & Severity	↑	(Shoar et al., 2020; Rathore et al., 2021)
	CK	Mortality & Severity	↑	(Parohan et al., 2020; Li et al., 2020c; Shoar et al., 2020; Malik et al., 2021; Zhu et al., 2021)
	CK-MB	Mortality & Severity	↑	(Li et al., 2020c; Zhu et al., 2021; Dalia et al., 2021; Alzahrani and Al-Rabia, 2021; Zinellu et al., 2021c)
	Myoglobin	Mortality & Severity	↑	(Parohan et al., 2020; Ma et al., 2021; Zhu et al., 2021)
	LDH	Mortality & Severity	↑	(Alnor et al., 2020; Zhang et al., 2020b; Kermali et al., 2020; Parohan et al., 2020; Li et al., 2020c; Shoar et al., 2020; Zheng et al., 2020; Suklan et al., 2021; Malik et al., 2021; Zhu et al., 2021; Hariyanto et al., 2021; Lim et al., 2021)
	IL6	Mortality & Severity	↑	(Alzahrani and Al-Rabia, 2021)
	CRP	Mortality & Severity	↑	(Alzahrani and Al-Rabia, 2021)
	HBDH	Mortality & Severity	↑	(Zinellu et al., 2021a)
	ApoA1	Mortality & Severity	↓	(Ulloque-Badaracco et al., 2021a)
	ApoB	Severity	↓	(Ulloque-Badaracco et al., 2021a)
**Liver Injury**	AST	Mortality & Severity	↑	(Alnor et al., 2020; Borges Do Nascimento et al., 2020; Toraih et al., 2020; Zheng et al., 2020; Ahmed et al., 2020; Abdulla et al., 2020; Shokri Afra et al., 2020; Aziz et al., 2020b; Suklan et al., 2021; Malik et al., 2021; Kovalic et al., 2021; Ye et al., 2021; Zarifian et al., 2021)
	ALT	Mortality & Severity	↑	(Borges Do Nascimento et al., 2020; Tian et al., 2020; Ahmed et al., 2020; Abdulla et al., 2020; Shokri Afra et al., 2020; Aziz et al., 2020b; Suklan et al., 2021; Malik et al., 2021; Ye et al., 2021; Zarifian et al., 2021)
	Albumin	Mortality & Severity	↓	(Tian et al., 2020; Aziz et al., 2020a; Kovalic et al., 2021; Hariyanto et al., 2021; Ye et al., 2021)
	Bilirubin	Mortality & Severity	↑	(Ahmed et al., 2020; Abdulla et al., 2020; Shokri Afra et al., 2020; Aziz et al., 2020b; Kovalic et al., 2021; Ye et al., 2021; Zarifian et al., 2021)
	LDH	Mortality & Severity	↑	(Alnor et al., 2020; Zhang et al., 2020b; Kermali et al., 2020; Suklan et al., 2021; Malik et al., 2021; Ye et al., 2021)
**Kidney Injury**	Creatinine	Mortality & Severity	↑	(Zheng et al., 2020; Tian et al., 2020; Malik et al., 2021)
	BUN	Severity	↑	(Vakhshoori et al., 2020a; Tian et al., 2020)
	Albumin	Mortality & Severity	↓	(Tian et al., 2020; Aziz et al., 2020a; Kovalic et al., 2021; Hariyanto et al., 2021; Ye et al., 2021)
**Lung Injury**	KL-6	Severity	↑	(Pramana Witarto et al., 2021; Naderi and Rahimzadeh, 2022)

*Reverse transcription polymerase chain reaction (RT-PCR), Point-of-Care (POC), immunoglobulin G (IgG), immunoglobulin M (IgM), immunoglobulin A (IgA), C-reactive protein (CRP), antiphospholipid antibodies (aPL), Type I interferons (IFN-I)-alpha (IFN-α), lactate dehydrogenase (LDH), aspartate aminotransferase (AST), alanine aminotransferase (ALT), Fibrinogen (fibrin) degradation products (FDPs), interleukin-4 (IL4), interleukin-6 (IL6), interleukin-8 (IL8), interleukin-10 (IL10), tumor necrosis factor (TNF), Creatine kinase (CK), brain natriuretic peptide (BNP), N-terminal proB-type natriuretic peptide (NT-proBNP), creatine kinase-myocardial bound (CK-MB), cardiac troponin T (TnT), cardiac troponin I (TnI), erythrocyte sedimentation rate (ESR), prothrombin time (PT), red blood cell distribution width (RDW), complement component 3 (C3), complement component 4 (C4), neutrophil-to-lymphocyte ratio (NLR), ApoliproteinA1 (ApoA1), ApoliproteinB (ApoB), blood urea nitrogen (BUN), von Willebrand Factor antigen (VWF-Ag), tissue-type plasminogen activator (t-PA), plasminogen activator inhibitor-1 antigen (PAI-1) antigen, soluble thrombomodulin (sTM), hydroxybutyrate dehydrogenase (HBDH), procalcitonin (PCT), Krebs von den Lungen-6 (KL-6), histo-blood group ABO system transferase (ABO), 2’-5’ oligoadenylate synthetase 1 (OAS1), white blood cell (WBC), mid-regional pro-adrenomedullin (MR-proADM), Vascular Cell Adhesion Molecule 1 (VCAM-1), Von Willebrand Factor Antigen (VWF-Ag), Angiopoietin-2 (Ang-2), tissue-type plasminogen activator (t-PA), plasminogen activator inhibitor-1 antigen (PAI-1) antigen, soluble thrombomodulin (sTM). "↑" refers to increased levels of specific biomarkers, while symbol "↓" refers to decreased levels of specific biomarkers listed in the Table 1.

Thus, several hematological and immunological markers could be included within the routine panel for SARS-CoV-2 infection evaluation to ensure risk stratification and effective disease management. It seems that the balance between an effective antiviral response and a dysregulated immune response is the key factor determining the severity of COVID19 progression. The levels of cytokines, especially interleukins (IL), in combination with T cell related immune signatures can be employed as biomarkers to predict COVID19 severity. Serum levels of IL2, IL2R, IL4, IL6, IL8, IL10 and TNFα were elevated in severe patients, with the largest differences observed for IL6 and IL10, as compared with the nonsevere COVID19 cases (Liu et al., 2021a; Jafrin et al., 2022). Furthermore, decreased levels of C3 and C4, indicative of complement activation, were associated with COVID19 severity and mortality and thus, could be used in predicting serious clinical outcomes in these patients (Zinellu and Mangoni, 2021b). Another systematic review on immunity and inflammatory biomarkers in COVID19 (Iwamura et al., 2021), demonstrated low counts of CD3+ and CD4+ T cells and lymphocytes, especially in severe and critical COVID19 patients, while an erythrocyte sedimentation rate (ESR), CRP and IL6 were elevated, independent of the severity of disease.

Recent evidence also indicated a potential role for calprotectin, a potential biomarker of inflammatory diseases, in identifying and stratifying COVID19 patients in terms of disease severity (Udeh et al., 2021). A systematic review and meta-analysis showed that calprotectin levels were significantly elevated in COVID19 patients who develop severe disease, suggesting its prognostic importance (Udeh et al., 2021). Increased homocysteine ​​levels were also recently suggested as a potential biomarker for predicting the risk of progression to serious COVID19 (Carpene et al., 2022). Presepsin was recently introduced as a potential new diagnostic biomarker for sepsis, with a high sensitivity and specificity (Zou et al., 2014). Based on findings from a recent systematic review (Ahmed et al., 2021b), elevated levels of presepsin appears to be another novel promising biomarker of COVID19 progression.

### 3.2 COVID19 mortality

It was reported that COVID19 patients who did not survive as compared with those who survived, had different levels of multiple biomarkers (**Table 1**), including elevated levels of cardiac troponin, CRP, IL6, D-dimer, creatinine and ALT as well as decreased levels of albumin (Tian et al., 2020; Elshazli et al., 2020). Recently, fibrinogen-to-albumin ratio and blood urea nitrogen-to-albumin ratio have been associated with adverse clinical outcomes of COVID19, including mortality (Ulloque-Badaracco et al., 2022). Another study found that lymphopenia and increased D-dimer levels were also associated with an increased risk of death (Figliozzi et al., 2020). Furthermore, it was demonstrated that lymphopenia, thrombocytopenia, increased WBC and platelet counts and elevated levels of D-dimer, CRP, procalcitonin (PCT), creatinine, and creatine kinase (CK), AST, ALT, and LDH activity were associated with higher risk of poor outcomes and deterioration of disease, including an intensive care admission, oxygen saturation <90%, invasive mechanical ventilation utilization and mortality (Malik et al., 2021; Shi et al., 2021; Xiang et al., 2021b).

In addition, procalcitonin (PCT) is recognized as a novel biomarker for early detection of systemic infections (Wacker et al., 2013) and its elevated serum levels also emerged as an additional prognostic factor for SARS-CoV-2 infection (Huang and Pranata, 2020; Hariyanto et al., 2021). Levels of PCT were elevated to varying degrees in severe and critical cases of COVID19 (Zheng et al., 2020; Ahmed et al., 2021a; Iwamura et al., 2021; Malik et al., 2021; Melo et al., 2021). Several systematic reviews and meta-analysis showed that PCT has good accuracy for the prognosis of severity and mortality in COVID19, and suggested that it can be considered as a single prognostic biomarker for adverse outcomes, including mortality (Zare et al., 2020; Malik et al., 2021; Melo et al., 2021).

Thus, as summarized in the **Table 1**, an abnormal levels of hematologic parameters, and other laboratory markers of hepatic function, inflammation, coagulation, and cardiac injury were associated with fatal outcome in COVID19 patients.

## 4 Biomarkers of injury of organ systems in COVID19

### 4.1 Immune system manifestations

An uncontrolled release of proinflammatory mediators in the form of cytokine storm by activated immune cells has been reported in COVID19 patients which contributes to an aberrant systemic inflammatory response and to severe pathological features observed in this disease. A recent systematic review with meta-analysis (Udomsinprasert et al., 2021) demonstrated an association of increased circulating levels of inflammatory cytokines, including IL6 and IL10, with the severity and mortality of COVID19 (**Table 1**). Another systematic review with meta-analysis also demonstrated a high level of circulating IL6 associated with the severity of infection by human coronaviruses strains, including SARS and MERS, while it was suggested that IL8, IL10, and TNF associate with the severity of SARS-Cov-2 infection only (Zawawi et al., 2021). Furthermore, recent systematic reviews (Chaudhary et al., 2021; Melo et al., 2021) pointed to increased levels of IL6, CRP, procalcitonin, D-dimer, ferritin, neutrophils and leucocytes associated with severe and fatal COVID19 cases. Another recent meta-analysis demonstrated about 3-fold higher levels of IL6 in patients with severe COVID19 as compared with patients with nonsevere disease (Coomes and Haghbayan, 2020), suggesting that the inhibition of IL6 may represent a potential novel target in COVID19 treatment.

A recent study also reported a difference in levels of IFN-α representing Type I interferons (IFN-I), a group of cytokines with an important function in antiviral responses, between patients with mild COVID19 and healthy individuals (Da Silva et al., 2021). However, there was no significant difference in plasma levels of IFN-α when comparing mild and severe patients (Da Silva et al., 2021), indicating that peripheral IFN-α can be potentially used as a marker of SARS-CoV-2 infection, but not as severity marker for COVID19.

Furthermore, it was indicated that leukocytosis and increased CRP levels on admission may predict severe outcomes, while leukopenia was associated with a better prognosis in patients with COVID19 (Yamada et al., 2020). High levels of CRP were found in patients with severe progress of COVID19 in which several organ systems were affected and in patients who died (Mosquera-Sulbaran et al., 2021). CRP activates complement, induces the production of pro-inflammatory cytokines and induces apoptosis which, together with the inflammatory state during the disease, can lead to a severe outcome. The results of recent systematic reviews (Ikeagwulonu et al., 2021; Yitbarek et al., 2021), comprised of more than 10,000 COVID-19 patients, confirmed the association of high levels of CRP with COVID19 severity, suggesting that COVID19 cases should be screened regularly for CRP levels to closely monitor disease severity and disease progression.

Furthermore, high serum amyloid A (SAA) levels have been also reported in SARS-CoV-2 infection (Kermali et al., 2020). It has been reported that SAA has a role as a cytokine-like protein in immunologic and inflammatory pathways (Sack, 2018) and as compared to CRP, it seems to be responsive to both viral and bacterial infections (Yip et al., 2005). The erythrocyte sedimentation rate (ESR) was also demonstrated to be elevated in COVID19 patients, regardless of disease severity (Iwamura et al., 2021).

It was also recently suggested that serum prealbumin, the combined biomarker of inflammation and malnutrition, might also be a potential marker for early risk stratification in COVID19 patients. A systematic review and meta-analysis showed that serum levels of prealbumin were decreased in patients with severe disease and in nonsurvivors, and its levels were negatively associated with age and CRP levels (Zinellu and Mangoni, 2021c).

In addition, several diagnostic strategies are employed to identify current SARS-CoV-2 infection or to test for past infection and immune response by using antibody tests (**Table 1**). A recent systematic review (Deeks et al., 2020) which included data for IgG, IgM, IgA, total antibodies and IgG/IgM from Asian, European, and USA study cohorts, showed low sensitivity during the first week since the beginning of symptoms, increasing in the second week and reaching their highest levels in the third week. Thus, it appears that antibody tests are expected to have a beneficial role for detecting previous SARS-CoV-2 infection if used after two weeks since the onset of symptoms, while there are limited data beyond 35 days post-symptom period (Deeks et al., 2020). According to an additional recent systematic review with meta-analysis, the detection of anti-SARS-CoV-2 IgM and IgG antibodies may assist in the early detection of SARS-CoV2 infection (Fathi et al., 2021), while IgA production may predict more severe COVID19 (Rangel-Ramirez et al., 2022). Furthermore, a previous study also showed a high prevalence of antiphospholipid antibodies (aPL) in patients with COVID19, with a recent meta-analysis and systematic review reporting that the most frequent type of aPL was lupus anticoagulant (LA) (Taha and Samavati, 2021). The authors also demonstrated higher prevalence of aPLs, anticardiolipin (aCL) (IgM or IgG) and anti-ß2 glycoprotein (anti-ß2 GPI) (IgM or IgG) antibodies in serious cases as compared with no critically ill patients (Taha and Samavati, 2021). Since the sensitivity of these antibody tests has mainly been evaluated in hospitalized patients, further studies are needed to understand whether these tests are able to detect lower antibody levels observed in milder and asymptomatic COVID19 disease.

It is also interesting to note that patients with a poor COVID19 prognosis demonstrated lower serum levels of vitamin D as compared with those with good prognosis (Munshi et al., 2021), which is in line with an immune modulator function of this vitamin. Thus, it was suggested that analysis of vitamin D levels could help in assessing potential development of severe COVID19, so that appropriate preventative and/or therapeutic interventions may be taken to improve COVID19 outcomes (Munshi et al., 2021).

#### 4.1.1 Pediatric multisystemic inflammatory syndrome

Since the beginning of the pandemic, COVID19 in children demonstrated a milder form and a better prognosis than in adults, and they are also likely to have a higher proportion of asymptomatic SARS-CoV-2 infection than adults (9) (Yasuhara et al., 2020; Chua et al., 2021). A systematic review on the clinical characteristics of COVID19 in children (Yasuhara et al., 2020), showed that the main clinical features were mild symptoms including fever, cough, and rhinorrhea, with lymphopenia and increased D-dimer and CRP levels (Yasuhara et al., 2020). However, although pediatric patients are generally mildly affected, it was demonstrated that infants might become seriously ill and some older children might develop the pediatric multisystemic inflammatory syndrome (PMIS) (Momtazmanesh et al., 2020; Yasuhara et al., 2020).

The PMIS is a severe, heterogeneous disease, affecting mostly previously healthy children and adolescents infected by SARS-CoV-2, with epidemiological enrichment demonstrated for males, adolescents, and ethnic minorities (Hoste et al., 2021). It is presenting with Kawasaki disease-like features and multiple organ failure, with fever, gastrointestinal and cardiovascular manifestations, respiratory symptoms and shock, and increased levels of inflammatory biomarkers (Yasuhara et al., 2020; Rodriguez-Gonzalez et al., 2020; Hoste et al., 2021; Tang et al., 2021). Recent systematic reviews reported that most of PMIS patients had increased levels of one or more inflammatory markers, including CRP, PCT, ESR, ferritin, IL6, and D-dimer, accompanied by neutrophilia and lymphopenia (Tang et al., 2021; Zhao et al., 2021c). Another systematic review found that these patients also exhibited higher B-type natriuretic peptide (BNP) levels than patients with nonsevere COVID19, while there was no significant differences in levels of another cardiac injury-specific biomarker, troponin (Zhao et al., 2021b).

### 4.2 Hematological manifestations

SARS-CoV-2 infection showed a substantial impact on the hematopoietic system and hemostasis as demonstrated by disturbance of levels of several laboratory parameters presented in **Table 1**. A systematic review and meta-analysis demonstrated that lymphopenia and thrombocytopenia were associated with serious outcomes in COVID19 patients (Huang and Pranata, 2020; Terpos et al., 2020; Lim et al., 2021; Pranata et al., 2021). It was also indicated that at a later phase of the disease, seven days since the onset of symptoms, lymphopenia aggravates while neutrophil count increases (Lim et al., 2021). A significant dose-response increase in levels of WBC and neutrophils was observed from nonsevere to severe progression and fatal outcomes (Chua et al., 2021). As shown in **Table 1**, severe COVID19 cases were also found to be associated with leukocytosis, neutrophilia, lymphopenia, and increased CK and LDH activity (Kovalic et al., 2021). Furthermore, neutrophil-to-lymphocyte ratio (NLR) and peak platelet/lymphocyte ratio were also suggested to have prognostic potential in identifying severe cases (Huang and Pranata, 2020). A recent systematic review with meta-analysis (Alkhatip et al., 2021) demonstrated higher NLR levels in COVID-19 patients as compared to SARS-CoV-2 negative subjects as well as in advanced COVID19 stages than in earlier stages (Alkhatip et al., 2021). According to additional recent systematic reviews with meta-analysis, higher NLR values were confirmed to be associated with the severity and mortality in hospitalized COVID-19 patients (Li et al., 2020b; Ulloque-Badaracco et al., 2021b; Alkhatip et al., 2021; Simadibrata et al., 2021; Sarkar et al., 2022a; Sarkar et al., 2022b; Parthasarathi et al., 2022), suggesting its use as a prognostic marker of COVID19.

Furthermore, iron metabolism also appears to have a significant role in the multiple organ dysfunction syndrome in COVID19. A systematic review with meta-analysis (Taneri et al., 2020) performed in about 57.000 patients diagnosed with COVID19 showed higher ferritin levels in nonsurvivors vs survivors (Taneri et al., 2020). Similar finding was also showed in other recent systematic reviews (Huang and Pranata, 2020; Huang et al., 2020; Kaushal et al., 2022) and it was suggested that hyperferritinemia should be considered as a red flag of systemic inflammation and a poor prognosis in COVID19 (Melo et al., 2021). In addition, emerging evidence emphasized the potential usefulness of measuring the red blood cell distribution width (RDW) to predict serious COVID19 outcomes. Higher levels of RDW were associated with COVID19 severity and mortality (Zinellu and Mangoni, 2021a; Lee et al., 2021). Thus, it was suggested that RDW could be utilized as a simple biomarker for early risk stratification in patients with SARS-CoV-2 infection (Lee et al., 2021).

In addition, higher protein expression of histo-blood group ABO system transferase (ABO) and lower protein expression of 2’-5’ oligoadenylate synthetase 1 (OAS1) were also found to be associated with higher risk of COVID-19 (Luo et al., 2022). The authors suggested that these proteomic signatures could also potentially help in assessing the progress and optimizing treatment for COVID19 (Luo et al., 2022).

#### 4.2.1 Blood coagulability

Blood hypercoagulability is another common finding among patients with COVID19 (Terpos et al., 2020), who seems to be at high risk for venous thromboembolism (VTE). A recent systematic review and meta-analysis demonstrated that mortality was higher in COVID19 patients with coagulopathy (Lim et al., 2020). It is not completely understood how aberrant fibrinolysis influences the clinical worsening of COVID19. Coagulation irregularities such as prothrombin time (PT) and aPTT prolongation, increased levels of fibrin degradation products, and severe thrombocytopenia could lead to life-threatening disseminated intravascular coagulation in COVID19 (Terpos et al., 2020). Furthermore, elevated levels of coagulation markers, such as PT, fibrinogen, fibrin and D-dimer may suggest the activation of coagulation pathways, thrombosis and the alarming progression of COVID19 to a potential serious outcome (Rostami and Mansouritorghabeh, 2020; Zhang et al., 2020a; Xiang et al., 2021a; Kovalic et al., 2021). Increased levels of D-Dimer might be an indicator of the occurrence of VTE in COVID19 patients (Liu et al., 2021b; Woller et al., 2022), while its steady increase during the disease course is particularly associated with serious disease progression and mortality (Terpos et al., 2020; Duz et al., 2020; Simadibrata and Lubis, 2020; Sakka et al., 2020; Lima et al., 2020; Huang et al., 2020; Paliogiannis et al., 2020; Ji et al., 2020; Varikasuvu et al., 2021; Nugroho et al., 2021; Chua et al., 2021). Thus, D-dimer is being considered as a key independent biomarker for the severity and mortality of COVID19 (Ji et al., 2020; Varikasuvu et al., 2021; Zhao et al., 2021a; Hariyanto et al., 2021).

In conclusion, several systematic reviews and meta-analyses confirmed that the levels of D-dimer, ferritin, neutrophil-to-lymphocyte ratio, and RDW have prognostic value in determining the severity and mortality of COVID19.

### 4.3 Endothelial dysfunction

Several studies also revealed the evidence on the key role of endothelial dysfunction in COVID19 progress. A recent meta-analysis showed that elevated levels of Mid-regional pro-adrenomedullin (MR-proADM), E-selectin, Vascular Cell Adhesion Molecule 1 (VCAM-1), Von Willebrand Factor Antigen (VWF-Ag), and Angiopoietin-2 (Ang-2) were associated with increased severity of this disease (Lampsas et al., 2021). Furthermore, another recent study also demonstrated increased levels of VWF-Ag in COVID19 patients (Andrianto et al., 2021). Levels of VWF-Ag, tissue-type plasminogen activator (t-PA), plasminogen activator inhibitor-1 antigen (PAI-1) antigen, and soluble thrombomodulin (sTM) were also reported to be associated with poor outcomes in COVID19 patients (Andrianto et al., 2021).

### 4.4 Cardiac injury

Although the respiratory and hematopoietic systems seem to represent the primary targets for SARS-CoV-2 infection, cardiovascular complications are emerging as additional serious outcomes in COVID19 that negatively impact patient prognosis and survival. It was found that an acute cardiac injury occurred in a significant number of COVID19 patients, leading to increased admission to the intensive care unit and higher mortality (Momtazmanesh et al., 2020; Vakhshoori et al., 2020b; Li et al., 2020a). Recently, Long et al. (Long et al., 2021) evaluated by meta-analysis the mortality risks associated with cardiovascular disease (CVD) and cardiac injury in hospitalized COVID19 patients in populations from four different countries and found that hospitalized COVID19 patients with cardiovascular events were at a higher risk of fatal outcomes than those without CVD (Shoar et al., 2020; Long et al., 2021).

In COVID19 patients admitted to the hospital, the incidence of heart failure, arrhythmias, acute myocardial injury and thrombotic events is high and often associated with disturbed levels of biomarkers of cardiac injury (Shoar et al., 2020; Pellicori et al., 2021). A recent systematic review reported that levels of cardiac and inflammatory markers were increased in 95 to 98% of patients with confirmed myocarditis due to COVID19 infection, respectively (Jaiswal et al., 2021). Based on that, cardiac injury-specific biomarkers are being used as prognostic tools in determining clinical outcomes and correlation to COVID19 disease severity. As shown in the **Table 1**, systematic reviews indicated that biomarkers such as cardiac troponin and N-terminal pro-B-type natriuretic peptide (NT-proBNP), as well as secondary markers, such as creatine kinase-myocardial bound (CK-MB), myoglobin, IL6, and CRP provided a prognostic tool in helping to early identify patients with the severe disease and that are susceptible to developing cardiovascular manifestations of COVID‐19 (Shafi et al., 2020; Alzahrani and Al-Rabia, 2021). Furthermore, cardiac injury, as assessed by higher serum levels of cardiac troponin I, LDH, myoglobin, CK, as well as cardiac troponin, IL6, CRP, and CK-MB were associated with the severity and death from SARS-CoV-2 infection, possibly due to immune-mediated myocardial injury (Shoar et al., 2020; Parohan et al., 2020; Li et al., 2020c; Walker et al., 2020; Alzahrani and Al-Rabia, 2021; An et al., 2021). As shown in **Table 1**, increased CK-MB levels were associated with severe disease and mortality in COVID19 patients (Alzahrani and Al-Rabia, 2021; An et al., 2021; Zinellu et al., 2021c; Qiang et al., 2021), suggesting that this biomarker of cardiac injury might be valuable for risk stratification in these patients (Zinellu et al., 2021c). It was also suggested that elevated levels of CK-MB in high-risk COVID19 patients can reflect an inflammation, organ damage, and prothrombotic predisposition as estimated by WBC count, AST & LDH activity, and D-dimer levels, respectively (Zinellu et al., 2021c). Interestingly, as shown in **Table 1**, all these biomarkers demonstrated significant associations with COVID19 severity (Paliogiannis et al., 2020; Zheng et al., 2020).

Although LDH has been known as a marker of cardiac injury, severe infections may lead to cytokine-mediated tissue damage and LDH release (Martinez-Outschoorn et al., 2011). Since LDH is present in lung issue, it can be expected that patients with severe SARS-CoV-2 infection release more LDH in their circulation. Previous studies reported that LDH could predict worse outcomes in hospitalized patients (Erez et al., 2014) and that its levels were shown to be increased in patients with Middle East Respiratory Syndrome (MERS) (Assiri et al., 2013). As shown in **Table 1**, a large number of systematic reviews and meta-analysis performed in COVID19 patients demonstrated elevated LDH levels (Alnor et al., 2020; Kermali et al., 2020; Li et al., 2020c; Parohan et al., 2020; Shoar et al., 2020; Zhang et al., 2020b; Zheng et al., 2020; Hariyanto et al., 2021; Lim et al., 2021; Malik et al., 2021; Suklan et al., 2021; Zhu et al., 2021), which seem to mirror the multiple organ injury and clinical consequences in these patients.

A previous meta-analysis demonstrated that increased troponin levels, found in about 30% of COVID19 patients, were associated with increased mortality in these patients (Wibowo et al., 2021). Additional systematic reviews reported that patients with increased troponin levels (Toraih et al., 2020; Vakhshoori et al., 2020b), when combined with either advanced age or elevated AST levels, were more likely to develop adverse outcomes (Toraih et al., 2020). Furthermore, high-sensitivity troponin I was also associated with increased severity and mortality in COVID19 patients (Chaudhary et al., 2021). A recent review showed that troponin and brain natriuretic peptide (BNP) were raised in almost 90% and 87% of SARS-CoV-2 infected patients, respectively (Rathore et al., 2021). Another study found that levels of high‐sensitivity troponin I (hsTnI) and NT-proBNP, increased during the course of hospitalization only in nonsurvivors (Li et al., 2020a). Recently, the cardiac outcomes were evaluated in about 52.000 patients and suggested that COVID19 is associated with persistent/*de novo* cardiac injury after recovery and with elevated levels of NT-proBNP (Ramadan et al., 2021). It was also demonstrated that higher BNP/NT-proBNP (B-type natriuretic peptide (BNP) or N-terminal proBNP (NT-proBNP) plasma levels were associated with severe disease (Zinellu et al., 2021b) and mortality (Pranata et al., 2020; Zinellu et al., 2021b) in COVID-19 patients. It appears that cardiac troponin T (TnT) had the highest odds ratio indicating the greatest association with COVID-19 severity and mortality, followed by NT-proBNP, cardiac troponin I (TnI), LDH, D-dimer, creatine kinase, and CK-MB (Qiang et al., 2021).

A recent systematic review and meta-analysis (Ma et al., 2021) performed in about 64.000 COVID19 patients found that elevated myoglobin levels was a more common finding than elevated cardiac troponin I (TnI) in patients with severe COVID19 and that elevated myoglobin levels were also associated with higher odds of severe illness and mortality than TnI. Thus, it was suggested that the increase of myoglobin levels may serve as an additional marker for predicting COVID19-related adverse outcomes (Ma et al., 2021).

Apolipoproteins, which are well established predictive biomarkers for cardiovascular and cerebrovascular diseases, have been recently proposed as prognostic biomarkers in infectious diseases, such as COVID19. Interestingly, it was reported that patients with low ApoliproteinA1 (ApoA1) and ApoliproteinB (ApoB) levels had a higher risk of developing severe COVOD19 (Ulloque-Badaracco et al., 2021a). Low ApoA1 levels were also associated with higher odds of all-cause mortality (Ulloque-Badaracco et al., 2021a). Thus, ApoA1 and ApoB can be employed as additional potential biomarkers for assessing the severity of COVID-19 (Ulloque-Badaracco et al., 2021a).

It was also reported that increased serum concentrations of hydroxybutyrate dehydrogenase (HBDH) were associated with COVID-19 severity and mortality, and suggested that this combined marker of myocardial and renal injury could also be used for risk stratification in COVID-19 (Zinellu et al., 2021a).

Therefore, cardiac injury-specific biomarkers, particularly cardiac troponin and myoglobin, may provide a useful prognostic tools in helping to recognize patients with the severe disease development or intensive care unit admission. It was suggested that they should be more frequently employed to identify high-risk COVID19 patients for developing COVID19 associated cardiomyopathy (An et al., 2021; Dy et al., 2021), so that timely interventions can be implemented to reduce the severity and mortality in COVID19 patients.

### 4.5 Kidney injury

An early evaluation and monitoring of both kidney and liver functions seems to be essential to forecast the progression of COVID19. Acute kidney injury (AKI) has been reported as a complication with high variability and controversial results. A systematic review and meta-analysis investigating COVID19 effects on renal function, demonstrated that AKI prevalence in COVID19 patients was 4% and it was significantly lower among nonsevere patients as compared to patients that did not survive (Vakhshoori et al., 2020a). It was also reported that AKI was associated with increased mortality, severe COVID19, and the need for ICU care (Lim et al., 2020). Nonsevere patients had lower blood urea nitrogen (BUN) levels as compared to deceased or those with severe SARS-CoV-2 infection (Vakhshoori et al., 2020a). Furthermore, levels of BUN, creatinine, and albumin were suggestive of kidney dysfunction at the time of admission in nonsurvivors as compared with survivors (Tian et al., 2020).

### 4.6 Liver injury

Several systematic reviews indicated an abnormal liver function in patients with COVID19 (Kovalic et al., 2021) (Sharma et al., 2021). Acute liver injury (ALI) was associated with increased severity and mortality in COVID19 (Lim et al., 2020; Sharma et al., 2021). A significant association between severe/critical SARS-CoV-2 infections with biochemical parameters, including elevated AST and ALT activity, and total bilirubin levels (Ahmed et al., 2020; Abdulla et al., 2020; Shokri Afra et al., 2020; Aziz et al., 2020b; Ye et al., 2021; Zarifian et al., 2021) and decreased albumin levels were noted (Ahmed et al., 2020; Abdulla et al., 2020; Kovalic et al., 2021; Ye et al., 2021; Zarifian et al., 2021). Furthermore, increased activities of liver enzymes were found to be more common in male patients with severe COVID19 than in their female counterparts (Shokri Afra et al., 2020). Increased AST, ALT, total bilirubin, and LDH levels and lower albumin levels also strongly correlated with COVID19 mortality (Ye et al., 2021). A recent systematic review and meta-analysis suggested that decreased albumin levels can be used for predicting severe COVID19 (Aziz et al., 2020a; Hariyanto et al., 2021). The previous studies indicated that COVID19 patients have a high prevalence of liver injury and the degree of the injury is associated with the severity of the disease (Abdulla et al., 2020; Ahmed et al., 2020; Shokri Afra et al., 2020; Ye et al., 2021), suggesting that disturbed levels of liver biomarkers, such as AST, ALT, bilirubin and albumin, could serve as prognostic tools in assessing the COVID19.

### 4.7 Lung injury

Lung injury is another common finding in COVID19 patients. Recent systematic reviews and meta-analysis showed that cough (53%) was one of mayor symptoms of COVID-19 as compared to muscle soreness (21%) and diarrhea (7%) (Wan et al., 2020). The severity of lung injury appears to be reflected in serum levels of Krebs von den Lungen-6 (KL-6) glycoprotein expressed on type II alveolar epithelium. Recent systematic reviews and meta-analysis found that high levels of serum KL-6 may depict more severe lung injury in COVID19 patients with moderately high sensitivity and specificity (Pramana Witarto et al., 2021). An additional systematic review and meta-analysis (Naderi and Rahimzadeh, 2022) also showed that serum levels of KL-6 were higher in severe COVID19 patients as compared to nonsevere and healthy subjects, suggesting the use of KL-6 as a potential biomarker for predicting severity of COVID19.

### 4.8 Brain manifestations

An increasing evidence demonstrates the presence of central and peripheral nervous system manifestations related to COVID-19, known as neuroCOVID (Leonardi et al., 2020). Abnormalities in an electroencephalogram (EEG) are common in COVID19-related encephalopathy and associate with disease severity, preexisting neurological conditions including prolonged EEG monitoring and epilepsy (Antony and Haneef, 2020). Frequent frontal findings have been proposed as a biomarker for COVID-19 encephalopathy (Antony and Haneef, 2020).

Developing acute ischemic stroke (AIS) significantly adds to the mortality of COVID-19 (Yassin et al., 2021). A recent systematic review and meta-analysis showed that the COVID-19+AIS group had higher lymphocytes, procalcitonin and creatinine levels (Yassin et al., 2021), suggesting that analysis of these potential biomarkers would be pertinent in predicting certain brain manifestations in COVID19 patients.

## 5 Conclusions

An increased evidence demonstrated the complexity of COVID19, with an unpredictable disease course that can rapidly progress to severe and deadly complications. The summary of published systematic reviews and meta-analyses that is presented in this paper, outlined the clinical significance of cardiovascular, respiratory and hepatic manifestations in the development of serious disease outcomes, while it was indicated that gastrointestinal and renal systems appeared not to be extensively affected in COVID19 patients. This summary highlighted the use of specific laboratory markers as the principal tools in assessing COVID19 progression. Based on the large body of evidence presented in this article, the specific biomarkers, including inflammatory and immunological parameters (CRP, PCT, IL6), hematological (lymphocyte and neutrophil counts, NLR, D-dimer, ferritin, RDW), cardiac (troponin, CK-MB, myoglobin), liver (AST, ALT, total bilirubin, albumin) and lung injury (KL-6), can be used as prognostic biomarkers that can aid the risk stratification and the prediction of serious clinical consequences, including mortality, in COVID19 patients. Potential novel biomarkers for COVID19 inflammatory and systemic manifestations also emerged recently, including procalcitonin, calprotectin and presepsin.

The majority of the findings presented here refer to the systematic reviews and meta-analyses including studies performed in hospitalized COVID19 patients. Thus, it would be pertinent to perform further studies in order to potentially dissect specific biomarkers whose levels change at the time of SARS-CoV-2 detection, before and during the symptoms onset, which would enable even earlier risk stratification, intervention, and prevention of potential serious outcomes in COVID19 patients.

## Author contributions

The author confirms being the sole contributor of this work and has approved it for publication.
